# ERK5 negatively regulates tobacco smoke-induced pulmonary epithelial–mesenchymal transition

**DOI:** 10.18632/oncotarget.3747

**Published:** 2015-04-17

**Authors:** Zhaofeng Liang, Wei Xie, Rui Wu, Hao Geng, Li Zhao, Chunfeng Xie, Xiaoting Li, Cong Huang, Jianyun Zhu, Mingming Zhu, Weiwei Zhu, Jieshu Wu, Shanshan Geng, Caiyun Zhong

**Affiliations:** ^1^ Department of Toxicology and Nutritional Science, Institute of Toxicology, School of Public Health, Nanjing Medical University, Nanjing, China; ^2^ Department of Surgery, The Second Affiliated Hospital of Anhui Medical University, Anhui, China; ^3^ The Key Laboratory of Modern Toxicology, Ministry of Education, School of Public Health, Nanjing Medical University, Nanjing, China

**Keywords:** tobacco smoke, lung cancer, ERK5, epithelial–mesenchymal transition, regulation

## Abstract

As the primary cause of lung cancer, tobacco smoke (TS) promotes the initiation and progression of lung tumorigenesis. Epithelial-mesenchymal transition (EMT) is a crucial process involved in cell malignant transformation. The role of ERK5, the lesser studied member of MAPKs family, in regulating TS-triggered pulmonary EMT has not been investigated. Normal human bronchial epithelial cells and BALB/c mice were used as *in vitro* and *in vivo* TS exposure models. Exposure of normal human bronchial epithelial cells to TS for 7 days induced morphological change, enhanced migratory and invasive capacities, reduced epithelial marker expression and increased mesenchymal marker expression. Importantly, we demonstrated for the first time that ERK5 negatively regulated TS-mediated lung epithelial EMT, as evidenced by the findings that TS suppressed ERK5 activation, and that TS-triggered EMT was mimicked with ERK5 inhibition and reversed by ERK5 overexpression. The negative regulation of ERK5 on pulmonary EMT was further confirmed in mice exposed to TS for 12 weeks. Taken together, our data suggest that ERK5 negatively regulates TS-mediated pulmonary EMT. These findings provide new insight into the molecular mechanisms of TS-associated lung tumorigenesis and may open up new avenues in the search for potential target of lung cancer intervention.

## INTRODUCTION

Tobacco smoke (TS) is causally associated with various types of cancers, especially lung cancer. Epidemiological and experimental studies have revealed the positive link between TS and risk of lung cancer development as well as other cancers [1–5]. It is estimated TS accounts for 80–90% of all lung cancer. Although enormous progress in understanding its molecular mechanisms leading to lung cancer development has been made, the molecular pathogenesis remains largely unknown [6].

Epithelial–mesenchymal transition (EMT) is a crucial pathophysiological process in embryonic development as well as cancer development [7–9]. During EMT process, cells lose their epithelial traits and acquire mesenchymal features. Evidences have suggested that, in addition to facilitating tumor invasion and metastasis, EMT is also critically involved in the initiation of tumorigenesis. Exposure of cells to carcinogens induces EMT during malignant transformation [10–14]. TS has been documented to promote EMT which regulates early events in lung carcinogenesis [13, 15–21]. Nonetheless, the underlying mechanisms by which TS induces EMT are poorly understood.

The mitogen-activated protein kinases (MAPKs) include four major subfamilies: extracellular regulated protein kinases 1 and 2 (ERK1/2), the Jun N-terminal kinases (JNKs), p38, and ERK5 [22, 23]. ERK5, also termed big MAPK1 (BMK1), is the least studied member of MAPKs family. The MEK5/ERK5 pathway is implicated in important cellular processes, including gene expression, proliferation, apoptosis, angiogenesis, cell motility and differentiation [24–27]. While some reports have suggested the functions of ERK5 in cancer oncogenesis, its role in EMT regulation has not been well explored. It has been shown that ERK5 promotes EMT in MCF-7, MDA-MB-468, MDA-MB-453 and SKBR3 breast cancer cells [28–30]. ERK5 triggers a motility and invasive phenotype of prostate cancer cells [31], osteosarcoma cells [32] and mesothelioma cells [33]. Importantly, several lines of evidence have suggested a differential regulatory role of ERK5 on EMT. Activation of ERK5 suppresses EMT in both Hs578T and MDA-MB-231 breast cancer cells [34]. ERK5 inhibits NF-κB activity, an essential step for EMT, in myoblast cells [35]. It has been reported that ERK5 positively regulates keratinocyte and vascular smooth muscle cell migration [36, 37], but negatively regulates hepatic and endothelial cell migration [38–40]. Therefore, ERK5 regulation of EMT may depend on cell types and/or cellular microenvironment.

To date, no studies have been done to examine the action of ERK5 in TS-induced lung epithelial EMT. Therefore, the present study was designed to investigate ERK5 regulation of TS-induced EMT. By using *in vitro* and *in vivo* TS exposure models, we demonstrate for the first time that ERK5 negatively regulated TS-mediated EMT. These novel findings indicate the important role of ERK5 activity in TS-associated carcinogenesis and may open new avenues in search for potential interventional target of TS-associated lung tumorigenesis.

## RESULTS

### TS induces EMT in normal human lung epithelial cells

**Figure 1:**
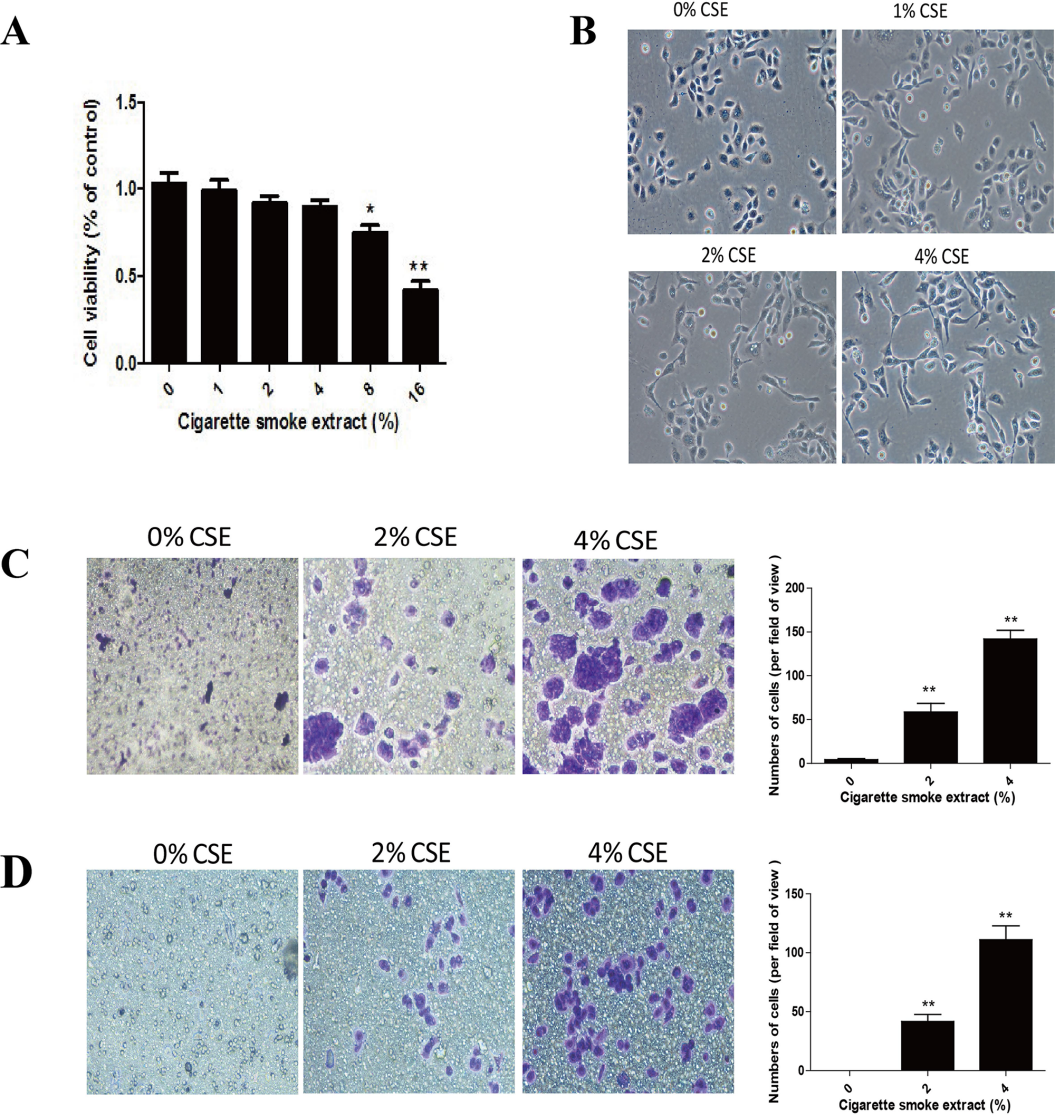
TS induces EMT morphological change and enhances migratory and invasive capacities of normal human bronchial epithelial (NHBE) cells **A.** Effect of cigarette smoke extract (CSE) on cell viability of NHBE cells. NHBE cells were treated with various concentrations of CSE for 7 days and cell viability was measured by MTT assay. **B.** CSE induced morphological change from epithelial to spindle-like mesenchymal shape, as shown by morphological examination of NHBE cells following CSE treatment for 7 days. **C.** CSE enhanced migratory capacity of NHBE cells, as determined by transwell migration assay. **D.** CSE enhanced invasive capacity of NHBE cells, determined by transwell invasion assay. Data are expressed as mean ± SD. **P < 0.01, compared with control group.

TS is the most important risk factor for lung cancer, and TS-induced EMT is critically involved in TS-associated malignant transformation. To investigate the effect of TS on EMT induction, NHBE cells were exposed to various concentrations of cigarette smoke extract (CSE) for 7 days. Cell viability was not apparently affected in cells treated with CSE at concentrations up to 4% (Figure 1A). Therefore, 4% CSE was selected as the maximum concentration for the following experiments.

**Figure 2:**
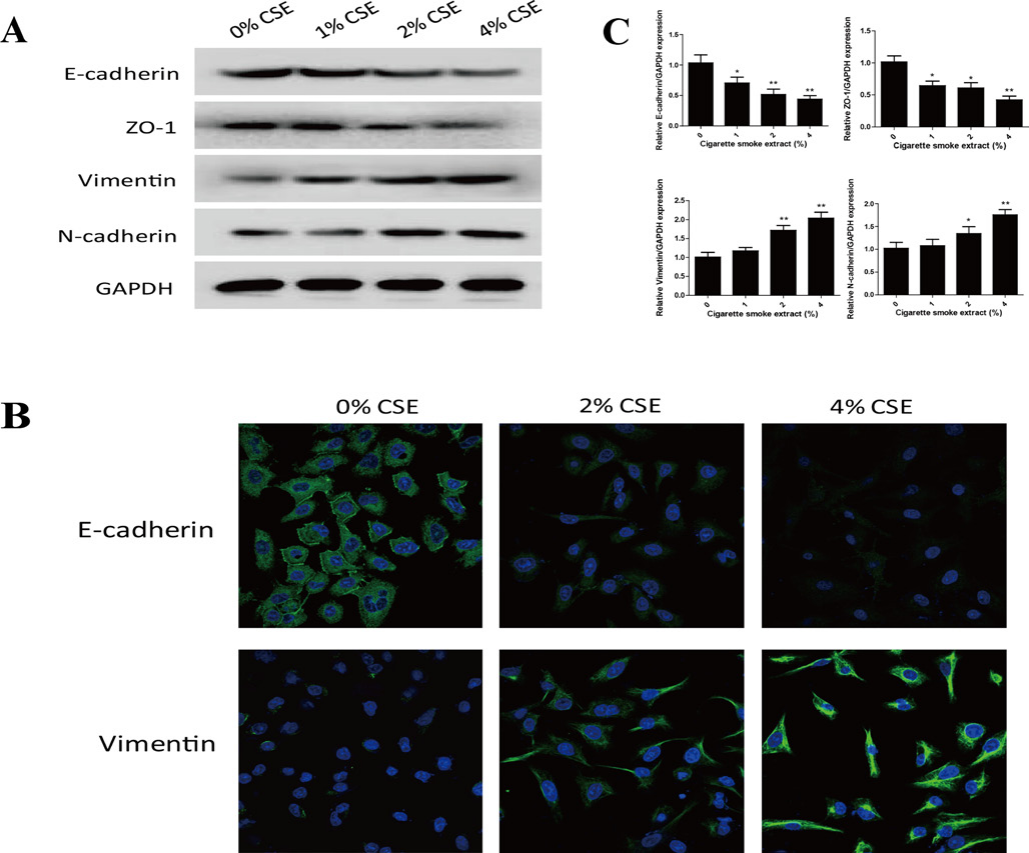
TS alters the expression of EMT markers in normal human bronchial epithelial (NHBE) cells **A.** Cigarette smoke extract (CSE) decreased the protein expression of epithelial markers E-cadherin and ZO-1, and increased protein expression of mesenchymal markers Vimentin and N-cadherin in NHBE cells, as shown by Western blotting. Glyceraldehyde 3-phosphate dehydrogenase was used as loading control. **B.** CSE decreased E-cadherin protein expression and increased Vimentin protein expression in NHBE cells shown by immunofluorescent staining. **C.** CSE decreased the expression of E-cadherin and ZO-1 mRNAs, and increased the expression of Vimentin and N-cadherin mRNAs, detected by quantitative reverse transcriptase–polymerase chain reaction after normalization to Glyceraldehyde 3-phosphate dehydrogenase. Data are expressed as mean ± SD. *P < 0.05, **P < 0.01, compared with control group.

EMT process is manifested by alterations in cell morphology, migration and invasion capacity, as well as expression of epithelial and mesenchymal markers. Treatment of NHBE cells with CSE for 7 days resulted in significant morphological change from epithelial round-shaped to spindle-like mesenchymal form (Figure 1B). To further examine the effect of TS on EMT, transwell assays were carried out to analyze NHBE cell migratory and invasive capacities inresponse to CSE. CSE treatment significantly increased NHBE cell migration (Figure 1C). Similarly, invasion of cells through reconstituted matrigel matrices was enhancedby CSE (Figure 1D). To determine whether molecular alterations of EMT occurred in CSE treated cells, the expression levels of EMT markers were determined. As measured by Western blot analyses, exposure of NHBE cells to CSE resulted in significant decreases in the protein expression of the epithelial markers E-cadherin and ZO-1. The levels of E-cadherin at 1% CSE, 2% CSE and 4% CSE were 95.61%, 62.09% (*p* < 0.05) and 40.96% (*p* < 0.01) of the control, respectively; the levels of ZO-1 at 1% CSE, 2% CSE and 4% CSE were 93.03%, 60.82% (*p* < 0.05) and 42.67% (*p* < 0.01) of the control, respectively. In contrast, CSE treatment significantly increased the protein levels of mesenchymal markers Vimentin and N-cadherin. CSE at concentrations of 1%, 2% and 4% caused 65.36% (*p* < 0.05), 93.67% (*p* < 0.01) and 180.54% (*p* < 0.01) increases in Vimentin, and −1.13%, 95% (*p* < 0.01) and 120.31% (*p* < 0.01) increases in N-cadherin, respectively (Figure 2A). Immunofluorescent staining also showed that CSE decreased E-cadherin protein expression and increased Vimentin expression in NHBE cells (Figure 2B). Moreover, qRT-PCR analyses revealed similar changes in the mRNA levels of epithelial and mesenchymal markers in NHBE cells exposed to CSE (Figure 2C). Collectively, data from morphological, migratory/invasive and molecular changes demonstrated that TS exposure induced EMT in human lung epithelial cells in a concentration-dependent manner.

### TS-induced pulmonary EMT is associated with ERK5 suppression

**Figure 3:**
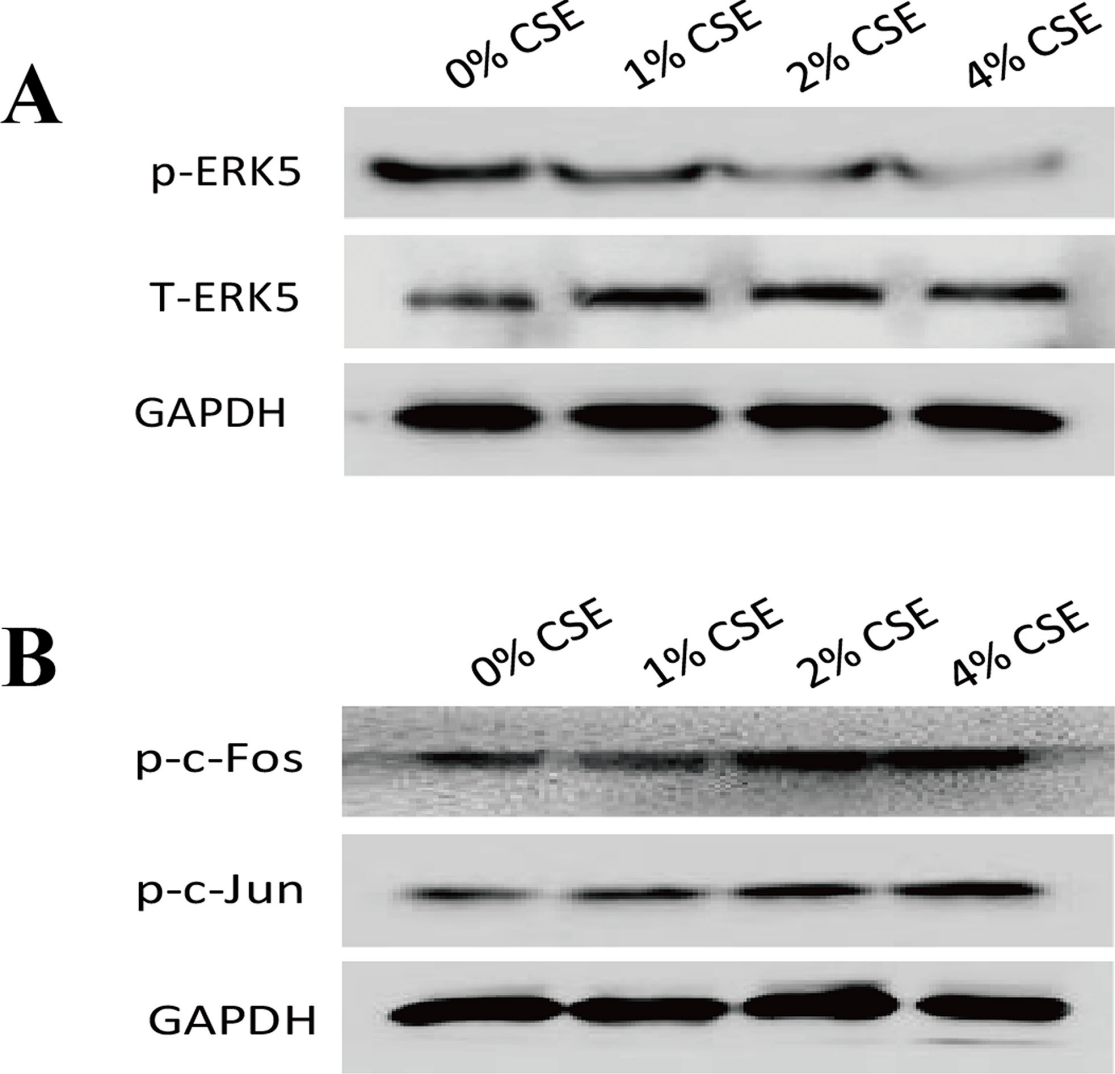
TS-induced EMT is associated with suppression of ERK5 activation in normal human bronchial epithelial (NHBE) cells **A.** Cigarette smoke extract (CSE) inhibited ERK5 activation in NHBE cells. The levels of phosphorylated ERK5, the active form of ERK5, as well as total ERK5, were measured by Western blotting following CSE treatment. **B.** CSE increased AP-1 protein activation, as shown by elevated levels of phosphorylated c-Fos and phosphorylated c-Jun. Glyceraldehyde 3-phosphate dehydrogenase was used as loading control.

As the lesser studied member of MAPKs family, ERK5 is implicated in cancer oncogenesis. The function of ERK5 in EMT regulation has not been well explored, and several lines of evidence have suggested that differential regulatory role of ERK5 on EMT may exist. To determine whether TS-elicited pulmonary cell EMT is associated with change in ERK5 activation, the level of phosphorylated ERK5, an indicator of ERK5 activation status, was measured. Exposure of NHBE cells to CSE for 7 days decreased phosphorylated ERK5 level. The levels of phospho-ERK5 at 1% CSE, 2% CSE and 4% CSE were 80.36%, 42.38% (*p* < 0.01) and 22.62% (*p* < 0.01) of the control, respectively. These results suggested the suppressive effect of TS on ERK5 activity. Meanwhile, CSE increased the activation of AP-1 protein in NHBE cells, as indicated by elevated levels of phosphorylated-c-Jun and phosphorylated c-Fos (Figure 3).

### Inhibition of ERK5 mimics TS-induced EMT in NHBE cells

**Figure 4:**
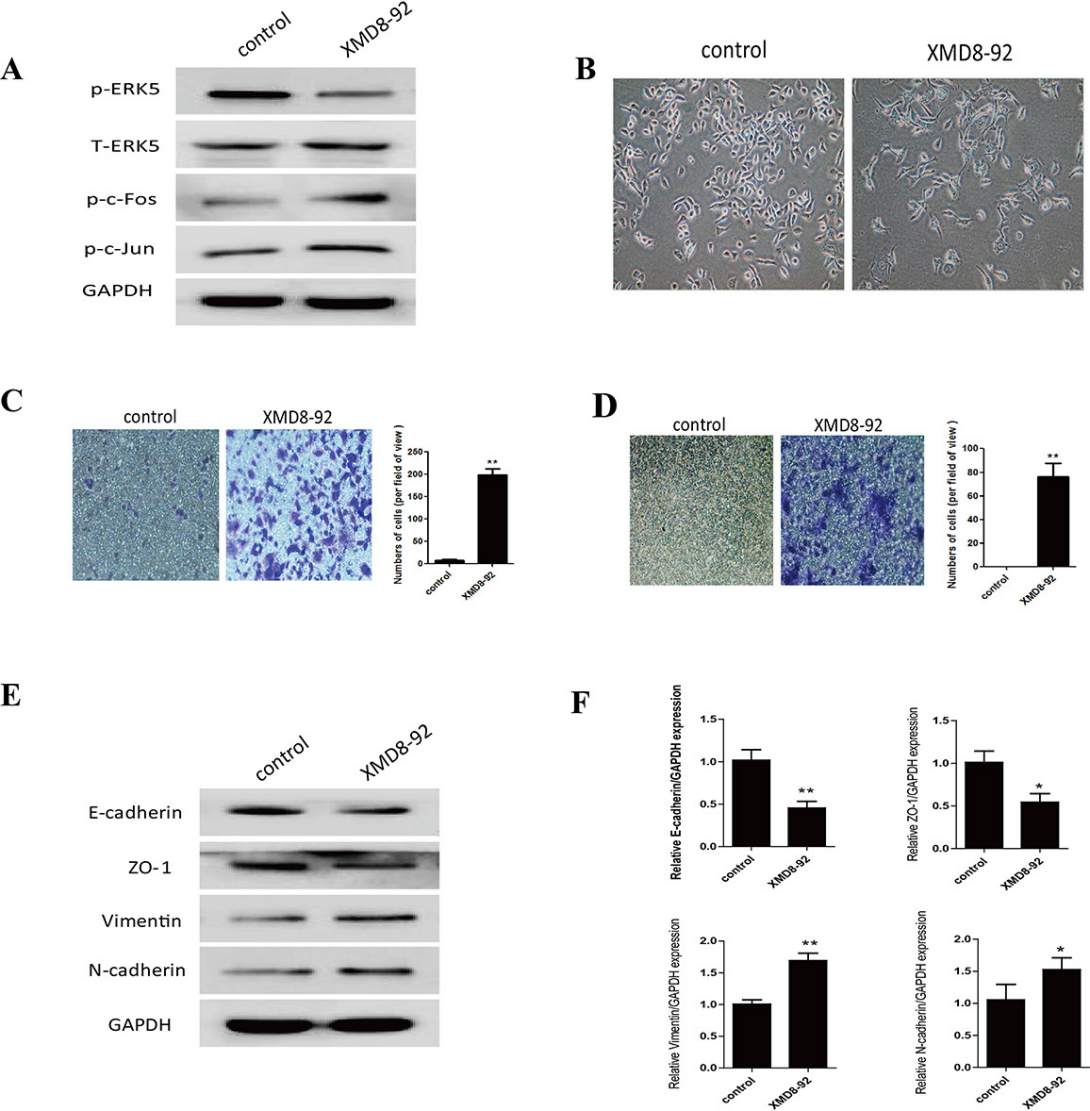
Inhibition of ERK5 mimics TS-induced EMT in normal human bronchial epithelial (NHBE) cells **A.** ERK5 inhibitor suppressed ERK5 activation in NHBE cells. NHBE cells were treated with a highly specific ERK5 inhibitor (XMD8-92) for 7 days, and Western blot analyses were performed for the measurements of phosphorylated ERK5, phosphorylated c-Fos and phosphorylated c-Jun. **B.** ERK5 inhibitor triggered morphological change of NHBE cells. **C.** ERK5 inhibitor enhanced migratory capacity of NHBE cells, as determined by transwell migration assay. **D.** ERK5 inhibitor increased invasive capacity of NHBE cells, as determined by invasion assay. **E.** Treatment of ERK5 inhibitor resulted in reduced protein levels of E-cadherin and ZO-1, and increased protein levels of Vimentin and N-cadherin. **F.** Treatment of ERK5 inhibitor decreased the expression of E-cadherin and ZO-1 mRNAs, and increased Vimentin and N-cadherin mRNAs. Data are expressed as mean ± SD. *P < 0.05, **P < 0.01, compared with control group.

Since above results revealed that TS-induced EMT was associated with ERK5 suppression in NHBE cells, we next explored the role of ERK5 in pulmonary EMT regulation. To mimic the inhibitory effect of TS on ERK5, NHBE cells were treated 7 days with 5 μM XMD8–92, a highly specific ERK5 inhibitor which suppresses ERK5 autophosphorylation and does not inhibit ERK 1/2 activity [24, 41]. As expected, XMD8–92 down-regulated phosphorylated ERK5 level and increased levels of phosphorylated-c-Jun and phosphorylated c-Fos in NHBE cells (Figure 4A). XMD8–92 treatment resulted in mesenchymal-like morphological change of the cells (Figure 4B). XMD8–92 also enhanced the migratory and invasive capacities of NHBE cells, as determined with transwell assays (Figure 4C, 4D). Furthermore, inhibition of ERK5 by XMD8–92 resulted in down-regulation of the protein and mRNA levels of the epithelial markers E-cadherin and ZO-1, as well as up-regulation of the mesenchymal markers Vimentin and N-cadherin; these effects were similar to that of TS on NHBE cells (Figure 4E, 4F). Thus, these results suggested that ERK5 activity may play an important role in CSE-induced EMT in NHBE cells.

### ERK5 overexpression reverses TS-triggered EMT of NHBE cells

**Figure 5:**
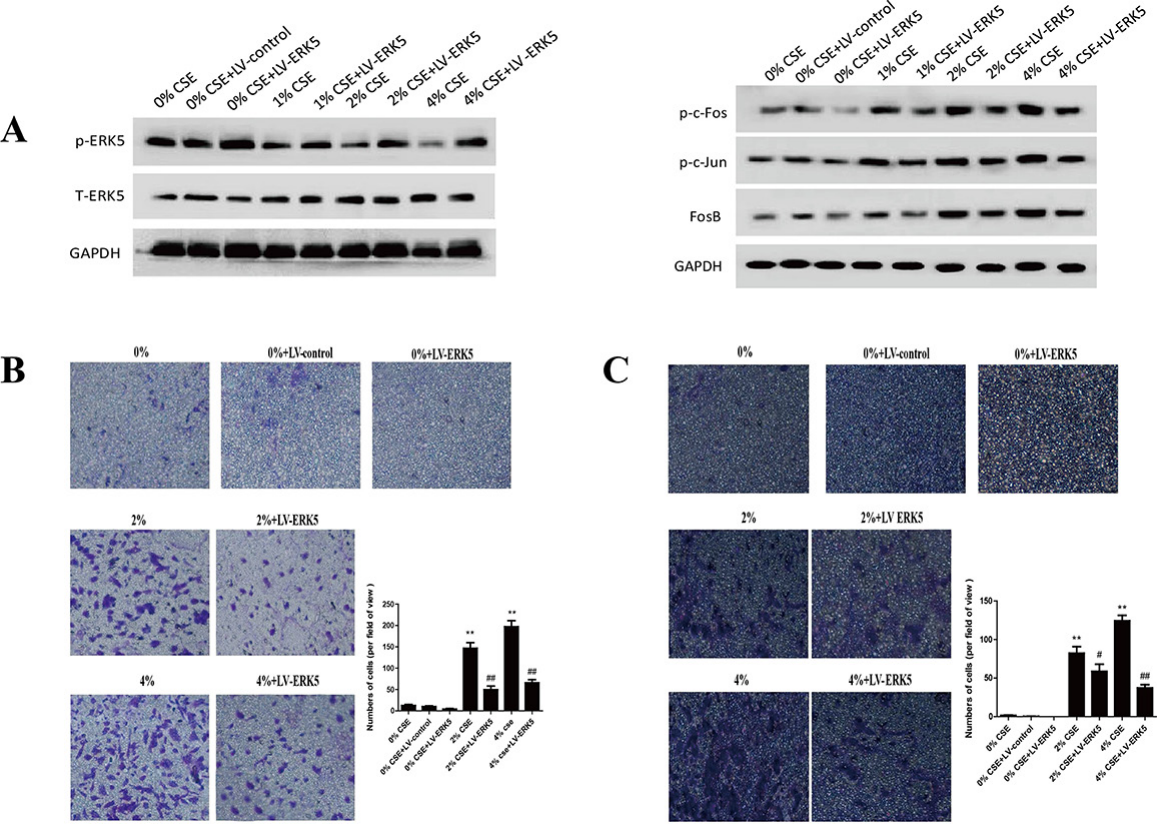
Overexpression of ERK5 reverses TS-triggered EMT morphological change and migratory and invasive capacities of normal human bronchial epithelial (NHBE) cells NHBE cells were stably transfected with lentiviral control vector or ERK5 expression vector at a multiplicity of infection of 25 for 72 hours, and then exposed to cigarette smoke extract (CSE) for 7 days. **A.** ERK5 expression vector restored CSE-suppressed ERK5 activity and attenuated CSE-induced activation of AP-1 subunits (c-Fos, c-Jun and Fos B) in NHBE cells, as measured by Western blotting. **B.** ERK5 overexpression reduced CSE-triggered migratory capacity of NHBE cells shown by migration assay. **C.** ERK5 overexpression decreased invasive capacity of NHBE cells as determined by invasion assay. LV-control = lentiviral control vector; LV-ERK5 = lentiviral expression vector for ERK5. *P < 0.05, **P < 0.01, compared with control group; ^#^*P* < 0.05, ^##^*P* < 0.01, compared with lentiviral control vector group.

**Figure 6:**
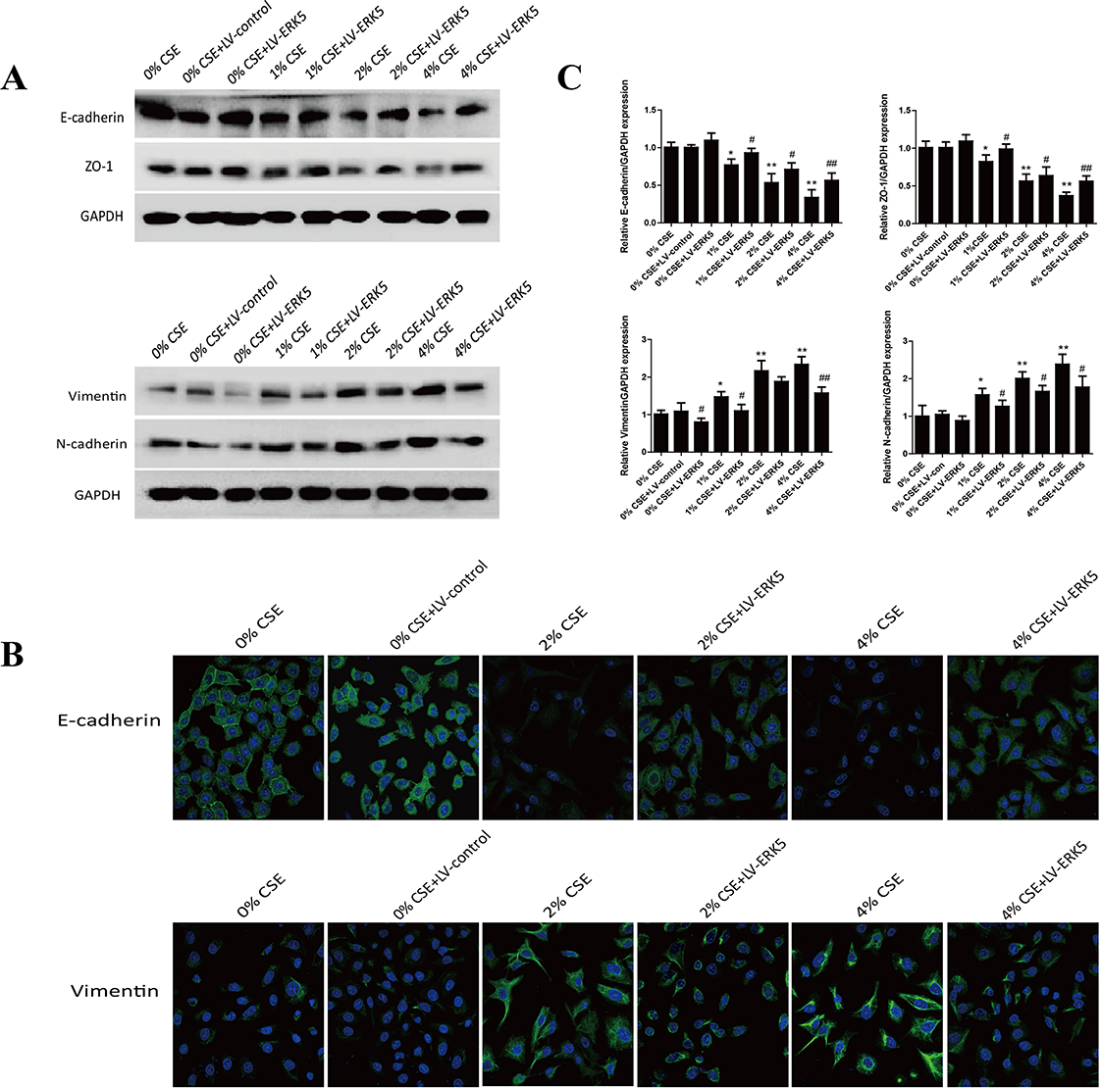
ERK5 overexpression attenuates TS-induced alterations in EMT markers' expression in normal human bronchial epithelial (NHBE) cells **A.** Transfection of ERK5 lentiviral vector in NHBE cells ameliorated cigarette smoke extract (CSE)-induced decreases in the protein expression levels of E-cadherin and ZO-1, as well as increases in protein expression levels of Vimentin and N-cadherin, as measured by Western blotting. **B.** ERK5 overexpression attenuated CSE-reduced E-cadherin protein expression and its increased Vimentin protein expression in NHBE cells, as shown by immunofluorescent staining. **C.** Overexpression of ERK5 improved CSE-induced alterations in the expression of E-cadherin, ZO-1, Vimentin and N-cadherin mRNAs. LV-control = lentiviral control vector; LV-ERK5 = lentiviral expression vector for ERK5. *P < 0.05, **P < 0.01, compared with control group; ^#^*P* < 0.05, ^##^*P* < 0.01, compared with lentiviral control vector group.

To further determine the role of ERK5 in TS-triggered EMT, NHBE cells were transfected with lentiviral ERK5 expression vector at multiplicity of infection (MOI) of 2.5, 5, 10, 25 and 50 for 72 hours. Green fluorescent protein (GFP) fluorescence imaging showed the successful transfection of NHBE cells with a MOI of 25 as the optimal infection efficiency. After transfection of ERK5 vector, NHBE cells were exposed to CSE for 7 days. Results showed that ERK5 expression vector restored CSE-suppressed ERK5 activity and prevented CSE-triggered activation of AP-1 subunits (c-Fos, c-Jun and Fos B) in NHBE cells (Figure 5A). Overexpression of ERK5 decreased CSE-mediated migration and invasion capacities of NHBE cells (Figure 5B, 5C). In addition, Western blot analyses showed that stable transfection of ERK5 with lentiviral vector attenuated CSE-induced decrease of E-cadherin and ZO-1 levels, as well as increase of Vimentin and N-cadherin in NHBE cells (Figure 6A). Immunofluorescent assay confirmed that transfection of ERK5 diminished CSE decreased E-cadherin expression and increased Vimentin expression in NHBE cells (Figure 6B). ERK5 overexpression ameliorated CSE-induced alterations in the mRNAs of E-cadherin, ZO-1, Vimentin and N-cadherin. Figure 6C Together, these data demonstrated the negative regulation of ERK5 on CSE-induced EMT in NHBE cells.

### TS alters EMT markers' expression and inhibits ERK5 activation in the lungs of mice

**Figure 7:**
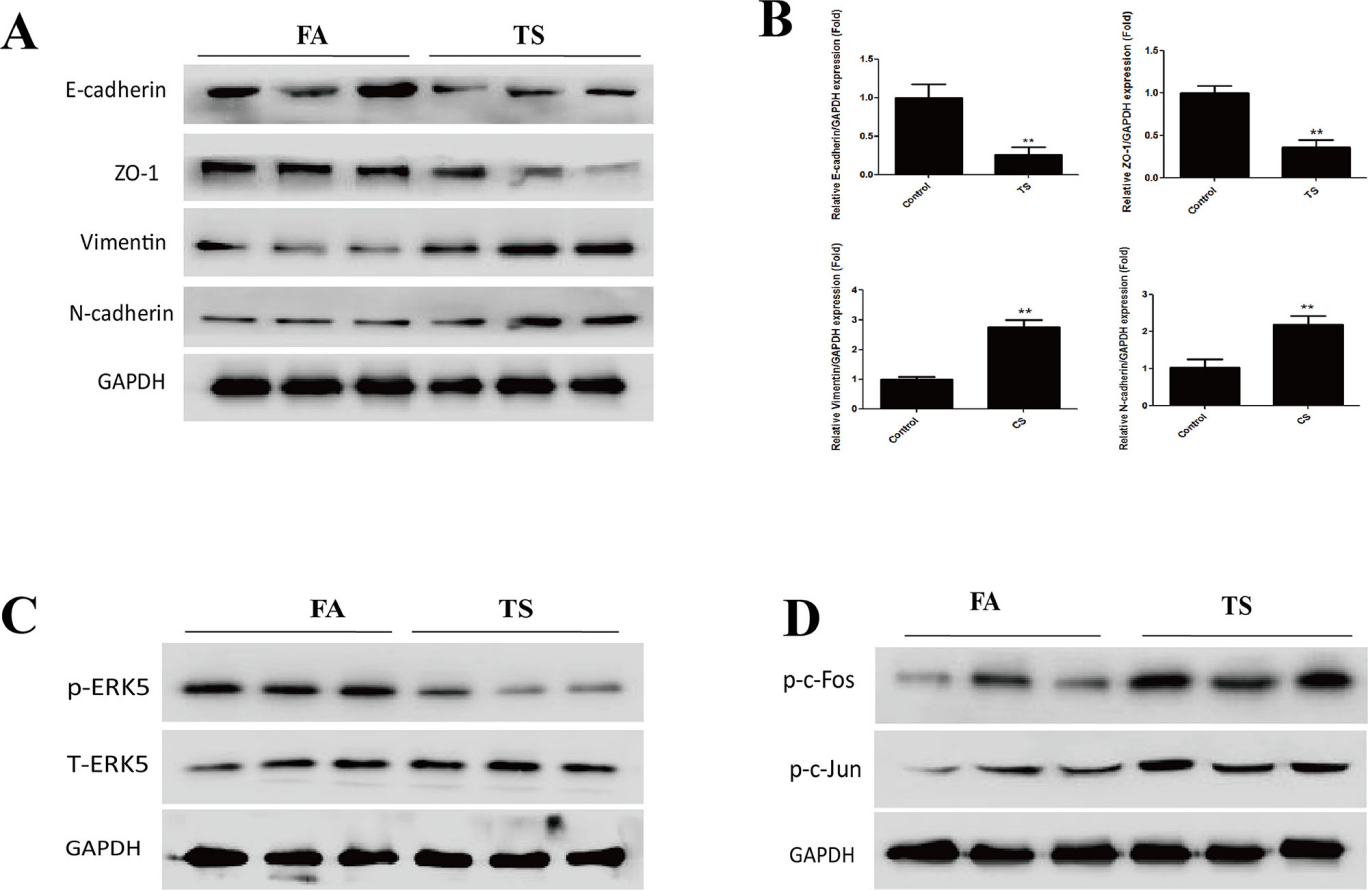
TS induces alterations in EMT markers' expression and ERK5 inhibition in the lungs of mice exposed to TS for 12 weeks **A.** TS decreased the protein levels of E-cadherin and ZO-1, and increased the protein levels of Vimentin and N-cadherin in the lungs of mice, as shown by. Western blotting. **B.** TS reduced mRNA levels of E-cadherin and ZO-1, and induced mRNA levels of Vimentin and N-cadherin in the lungs, detected by quantitative reverse transcriptase–polymerase chain reaction. **C.** TS inhibited ERK5 activation in mouse lungs shown by Western blot analysis of phosphorylated ERK5. **D.** TS elevated levels of phosphorylated c-Fos and phosphorylated c-Jun as assesed by Western blotting. Data are expressed as mean ± SD. **P < 0.01, compared with FA control group. FA = filtered air; TS = tobacco smoke.

Following the illustration of the negative regulatory function of ERK5 in CSE-induced pulmonary EMT *in vitro*, we further investigated whether TS induces EMT-like changes and modulates ERK5 activation in an animal model. Mice were exposed to TS for 12 weeks, and the expressions of the epithelial markers E-cadherin and ZO-1, as well as the mesenchymal markers Vimentin and N-cadherin in lung tissues were examined. We revealed that TS exposure significantly decreased protein and mRNA expressions of E-cadherin and ZO-1, and significantly elevated the expression of Vimentin and N-cadherin in the lungs. Western blot analyses showed that the levels of E-cadherin, ZO-1, Vimentin, and N-cadherin in TS exposed mice were 30.65% (*p* < 0.01), 41.96% (*p* < 0.01), 276.25% (*p* < 0.01), and 217.58% (*p* < 0.01) of the control, respectively (Figure 7A). qRT-PCR assays showed that TS induced changes in mRNA levels of these EMT markers were consistent with that of protein measurements (Figure 7B).

**Figure 8:**
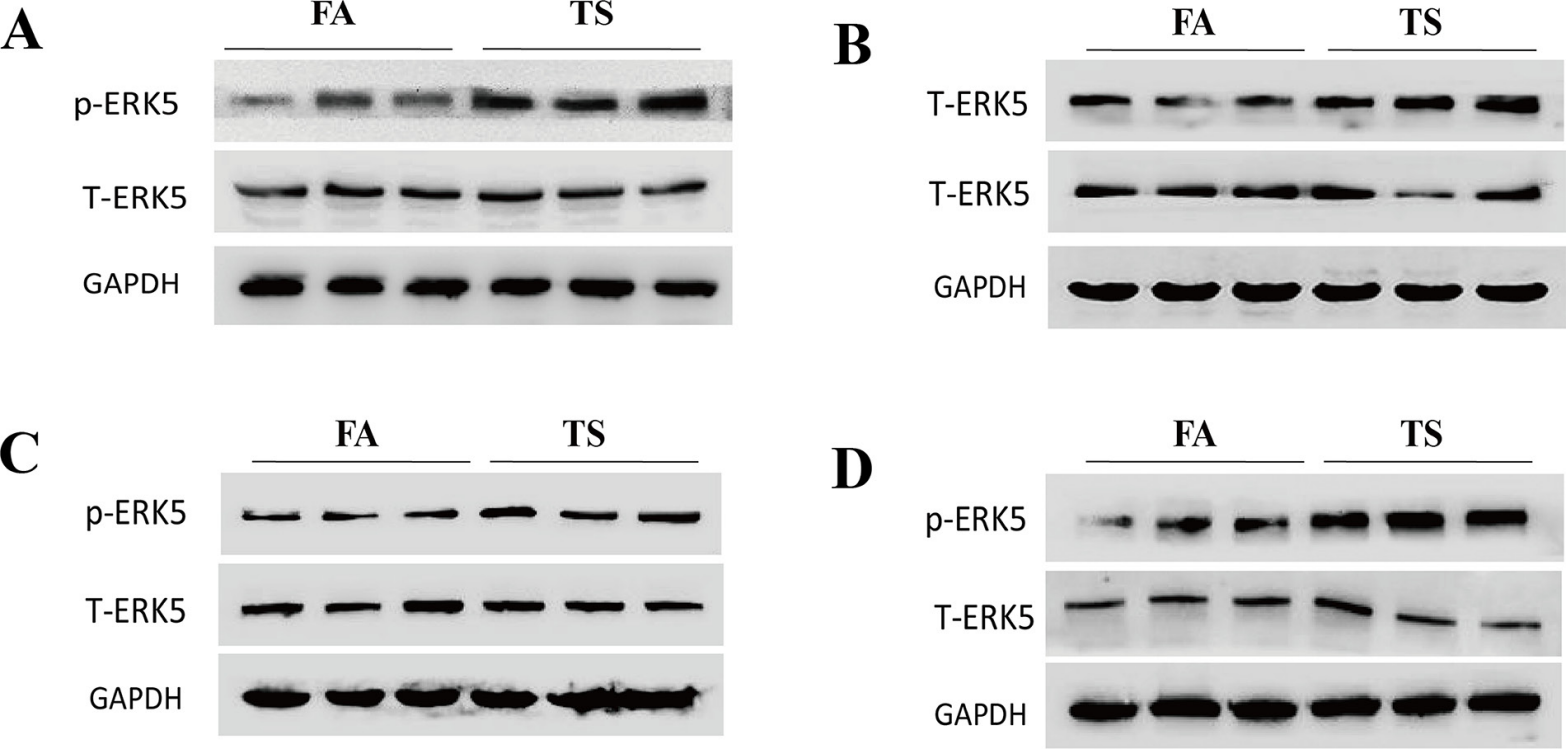
TS increases ERK5 activation in the stomach, liver, kidney and bladder of mice Exposure of mice to TS for 12 weeks elevated the activation of ERK5 in the tissues of stomach **A.** liver **B.** kidney **C.** and bladder **D.** as shown by Western blot analyses of phosphorylated ERK5. FA = filtered air; TS = tobacco smoke.

Similarly, in our study we found that exposure of mice to TS resulted in significant decreased ERK5 activity and increased c-Jun and c-Fos activation in lung tissues (Figure 7C and 7D). These data were in line with our *in vitro* findings. Intriguingly, we also analyzed ERK5 activation status in other tissues of mice exposed to TS in this study and observed that TS increased, rather than decreased, ERK5 activation in stomach, liver, kidney and bladder tissues of mice (Figure 8). These results suggest tissue specific modulation of TS on ERK5 activation.

### Ectopic overexpression of ERK5 attenuates TS-induced lung EMT alterations in mice

**Figure 9:**
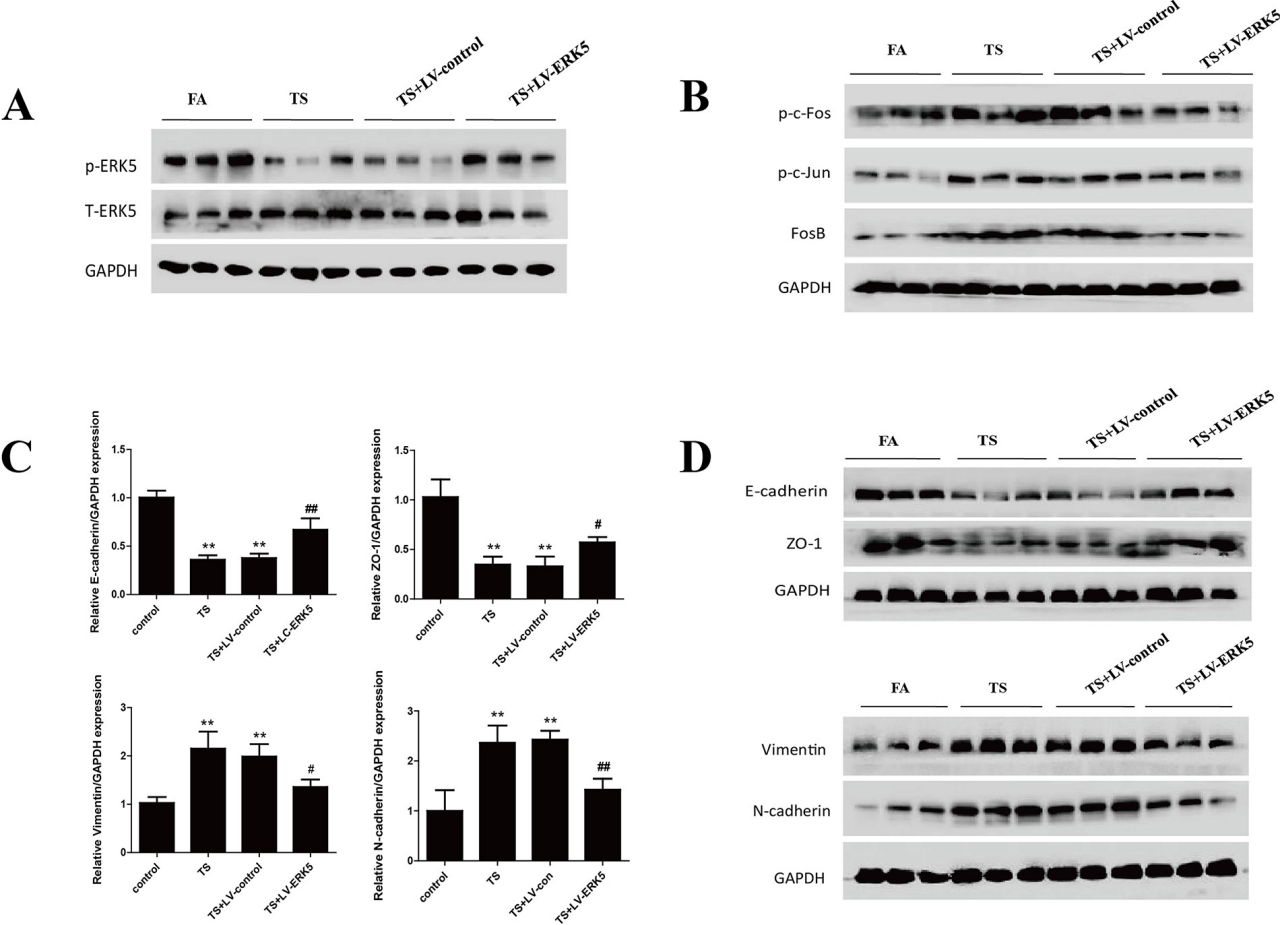
Overexpression of ERK5 reverses TS-induced pulmonary EMT alterations in mice **A.** Intratracheal delivery of lentiviral ERK5 expression vector restored ERK5 activation in the lung tissues of mice exposed to TS for 12 weeks, as shown by Western blotting. **B.** Lentivirus-mediated ERK5 overexpression suppressed TS-induced activation of c-Fos, c-Jun and FosB proteins in mouse lungs. **C.** Lentivirus-mediated ERK5 overexpression attenuated TS-induced alterations in mRNA expressions of E-cadherin, ZO-1, Vimentin and N-cadherin, as measured by quantitative reverse transcriptase–polymerase chain reaction. **D.** ERK5 overexpression prevented TS-induced alterations in protein expression of E-cadherin, ZO-1, Vimentin and N-cadherin, as determined by Western blotting. Data are expressed as mean ± SD. *P < 0.05, compared with FA control group; **P < 0.01, compared with FA control group; ^#^*P* < 0.05, compared with TS-LV-control group; ^##^*P* < 0.01, compared with TS-LV-control group. FA = filtered air; TS = tobacco smoke; LV-control = lentiviral control vector; LV-ERK5 = lentiviral vector for ERK5.

In order to investigate the negative regulation of ERK5 in TS-mediated EMT *in vivo*, mice were intratracheally delivered with lentiviral vector for ERK5 and exposed to TS for 12 weeks. Western blot analysis showed that TS-decreased ERK5 activation in lung tissues was restored by the delivery of ERK5 vector (Figure 9A). Ectopic expression of ERK5 decreased TS-induced activation of AP-1 subunits (c-Fos, c-Jun, FosB) (Figure 9B). Moreover, TS-induced alterations in protein and mRNA expression of the EMT markers, including decreases of the epithelial markers E-cadherin and ZO-1, and increases of the mesenchymal markers Vimentin and N-cadherin, were effectively attenuated with ERK5 vector delivery (Figure 9C and 9D). These data indicated that overexpressed ERK5 reversed TS-induced pulmonary EMT changes in mice.

## DISCUSSION

TS is the primary cause of lung cancer, which promotes the initiation and progression of lung tumorigenesis. The underlying molecular mechanisms by which TS causes lung cancer development remain to be established. In the present study we revealed that TS induced EMT in both NHBE cells and the lungs of mice. Most importantly, we demonstrated for the first time that ERK5 negatively regulated TS-mediated EMT, as evidenced by the findings that TS suppressed pulmonary ERK5 activation, and that TS-triggered EMT phenotypic alterations was mimicked with ERK5 inhibition and reversed by ERK5 overexpression. These findings suggest the important role of ERK5 activity in TS-associated EMT and provide critical information for the molecular mechanisms of TS-related lung tumorigenesis as well as search for the potential target of lung cancer intervention.

Characterized by changes in cell morphology, migration and invasion capacity, as well as expression prolife of epithelial and mesenchymal markers, EMT is a crucial process in cancer development. Evidences have revealed that exposure of cells to carcinogens induces EMT during transformation and tumor formation [10–14], suggesting the important role of EMT in the initiation of tumorigenesis by promoting cell malignant transformation. Tellez et al demonstrated that exposure of human bronchial epithelial cells to tobacco carcinogens benzo(a)pyrene-diolepoxide and methylnitrosourea induces EMT, which is an early manifestation during transformation process, participating in cancer initiation and promoting the clonal expansion of premalignant cells [10]. Evidences also show that EMT is involved in arsenite-induced transformation of human bronchial epithelial cells [11, 12]. It has been documented that TS promotes EMT, resulting in loss of cellular polarity, down-regulation of epithelial cadherin, loss of cell-cell adhesion, and increased mobility of lung epithelial cells [15–21]. In agreement with previous reports, we showed in the present study that exposure to TS induced EMT in NHBE cells, as manifested by morphological change from epithelial to mesenchymal form, increased migratory and invasive capacities, as well as alterations in the expression of EMT markers, including decreased epithelial markers E-cadherin and ZO-1, and increased mesenchymal markers Vimentin and N-cadherin. Moreover, we also showed that TS exposure resulted in decreased expression of E-cadherin and ZO-1, as well as increased expression of Vimentin and N-cadherin in the lungs of mice, suggestive of EMT induction. Taken together, our data revealed that TS triggered EMT in both *in vitro* and *in vivo* settings.

Several cell signaling factors have been implicated in EMT. Nonetheless, the underlying mechanisms of EMT induction by TS are poorly understood. ERK5 is the lesser studied member of the proto-oncogenic MAPKs family. Some reports have depicted the important biological functions of ERK5 in cancer oncogenesis; however, its role in EMT regulation has not been well characterized, and the available evidences suggesting the action of ERK5 in EMT process remain elusive. On one hand, it has been shown that ERK5 promotes EMT in MCF-7 breast cancer cells [28, 29] and facilitates migration and invasion of MDA-MB-468, MDA-MB-453 and SKBR3 breast cancer cells [30]. ERK5 triggers a motility and invasive phenotype of prostate cancer cells [31], osteosarcoma cells [32], and mesothelioma cells [33]. Upregulated ERK5 expression correlates with high-grade prostate cancer and oral squamous cell carcinoma [42, 43]. On the other hand, evidences have suggested a differential regulatory role of ERK5 on EMT. ERK5 activation results in significant decreased migration and invasion of both Hs578T and MDA-MB-231 breast cancer cells, whereas knockdown of ERK5 increases cell migratory and invasive capacities and Vimentin expression, indicating ERK5 as a negative regulator of EMT in these breast cancer cells [30]. ERK5 positively regulates keratinocyte and vascular smooth muscle cell migration [36, 37], but negatively regulates hepatic and endothelial cell migration [38–40]. These evidences suggest that ERK5 regulation of EMT may be cell types and/or cellular microenvironment sensitive.

To date, no studies have been done to investigate the function of ERK5 in TS-induced EMT or in lung cancer cell invasion and metastasis. In the present study we showed that TS-induced pulmonary EMT was associated with suppression of ERK5 activation *in vitro* and *in vivo*. To determine the role of ERK5 in pulmonary EMT regulation, the inhibitory effect of TS on ERK5 was mimicked with a highly specific ERK5 inhibitor. Inhibition of ERK5 alone resulted in effects similar to that of TS on NHBE cells, including mesenchymal-like morphological changes, enhanced migratory and invasive capacities, and alterations in EMT markers expression. Furthermore, we illustrated that overexpression of ERK5 reversed TS-triggered EMT in both NHBE cells and the lungs of mice. Collectively, these data clearly indicated that ERK5 negatively regulated TS-induced pulmonary EMT.

Intriguingly, in the present study we also analyzed ERK5 activation status in other tissues of mice exposed to TS. We observed that TS increased, rather than decreased, ERK5 activity in other tissues such as stomach, liver, kidney and bladder of mice. These results provided evidence for the tissue sensitivity of ERK5 activation regulated by TS. In addition, we compared ERK5 activation in NHBE cells with non-small cell lung cancer cells and found reduced ERK5 activity in lung cancer cells (unpublished data). In supporting our finding is the observation of low ERK5 activation in H2122 and H157 non-small cell lung cancer cells in comparison with high level of ERK1/2 activation [44]. Based on these findings from the present study in non-malignant lung cells, investigation on the distinct role of ERK5 in regulating EMT and invasion and metastasis in malignant transformed lung cancer cells is ongoing.

As the proto-oncogenic signaling, MAPKs pathways are generally thought to promote cancer development; while differential biological functions may exist for individual MAPK pathways. Among MAPKs family, ERK5 is twice the size of other members. The N-terminal half of ERK5 contains the kinase domain which is similar to that of ERK1/2 and has the TEY activation motif. Unlike other MAPKs, the C-terminal region of ERK5 contains a long non-catalytic domain which has a unique function. Upon activation, ERK5 phosphorylates and activates downstream target molecules, including transcription factors such as members of the AP1 proteins. Activation of ERK5 also results in autophosphorylation of the C-terminus, which alone has the ability to increase transcription activity. Therefore, ERK5 differs from other ERKs in possessing transcriptional activation activity. It is able to regulate the transcription of downstream molecules in two ways, i.e., through either the phosphorylation or the enhancement of the transcription activity of target molecules [24–27]. Nevertheless, only a small number of molecules have been identified as the target genes of ERK5. Notably, it has been found that ERK5 transcriptional regulates gene expression in a tissue-specific manner [45]. In skeletal muscle cells, activation of ERK5 significantly inhibits TNF-α-mediated NF-κB activity [35]. In lung microvascularendothelial cell, activation of ERK5 suppresses HIF1alpha expression and inhibits angiogenesis [38]. In this study we showed that TS-mediated pulmonary ERK5 inhibition increased the activation of AP-1 proteins c-Fos and c-Jun; on the contrary, overexpressed ERK5 decreased c-Fos and c-Jun activation, suggesting the negative modulation of ERK5 on AP-1. Evidences have suggested that AP-1 proteins play key role in EMT regulation by increasing the expression of master switch EMT-inducing transcription factors such as Snai1, Slug, Zeb1/2, Twist1/2, and TGFβ, which in turn transcriptionally increase the expression of mesenchymal markers Vimentin and N-cadherin, and suppress the expression of epithelial markers E-cadherin and ZO-1 etc. In addition, AP-1 can directly regulate the transcription of mesenchymal and epithelial markers involved in EMT induction [46–49]. It is noteworthy that the precise mechanism by which ERK5 regulates AP-1 activation is unknown at present time, and future studies are warranted to define the underlying mechanism of pulmonary ERK5 regulation on AP-1 protein.

ERK5 activity is regulated by its phosphorylation and dephosphorylation status [24–27]. It has been demonstrated that ERK5 is activated in response to a variety of stimuli upon phosphorylation by its upstream kinase MEK 5, which specifically recognizes the TEY motif in the N-terminal half, whereas the phosphotyrosine-specific phosphatase PTP-SL (protein tyrosinephosphatase STEP-like) is able to dephosphorylate ERK5, thereby hindering its translocation to the nucleus [50]. Further regulation of ERK5 by post-translational modifications has been illustrated. A small ubiquitin-like modifier 3 (SUMO3) protein covalently binds to ERK5 on Lys6 and Lys22 and inhibits its transcriptional promoter activity without affecting ERK5 phosphorylation [51]. Additionally, it has been observed that microRNAs (miRNAs) are able to regulate ERK5 activity. Expression of miR-143 and miR-145 has been shown to down-regulate levels of ERK5 [52–53]. Scapoli L et al. has demonstrated that activation of Src, the non-receptor tyrosine kinase, is involved in ERK5 phosphorylation and activation in murine alveolar epithelial type II cell line caused by EGF, H_2_O_2_ or asbestos treatment for up to 24 hours [54]. A recent study has shown that Src activation is involved in tobacco smoke induced EMT in transformed lung cancer cells with 72 hours treatment [55]. In the present study we have showed that tobacco smoke decreases the level of phosphorylated ERK5 which negatively regulates EMT in normal human bronchial epithelial cells with 7 days treatment and in normal mouse lungs with 12 weeks treatment. It is possible that Src activation by tobacco smoke may be involved in the regulation of ERK5 activity and EMT, although the experimental conditions including *in vitro* and *in vivo* models as well as treatment durations were different in our study from that of other studies. Other post-transcriptional modifications may also participate in TS induced regulation of ERK5 activation.

In summary, the present study demonstrates for the first time that ERK5 negatively regulates TS-induced pulmonary EMT in both *in vitro* and *in vivo* settings. These novel findings indicate the important role of ERK5 activity in tobacco smoke-related carcinogenesis and provide a plausible strategy for the search of potential interventional target of tobacco smoke-associated lung tumorigenesis.

## METHODS

*See* the supplementary material for further details concerning methods.

### Culture and smoke treatment of NHBE cells

Normal human bronchial epithelial (NHBE) cells were purchased from XiangYa Central Experiment Laboratory (Changsha, China) and cultured in RPMI 1640 Medium. Cigarette smoke extract (CSE) was prepared daily immediately before use according to the reported method [56–58]. One filterless Research cigarette (3R4F) was combusted and used for preparation of 10 ml medium which was referred to as a 100% CSE solution. NHBE cells were exposed to various concentrations of CSE for 7d.

### Transwell assays

Migratory and invasive capacities of CSE-treated NHBE cells were evaluated using Transwell chambers without or with matrigel, respectively.

### Immunofluorescent staining

Immunofluorescent staining was performed to analyze the expression of E-cadherin and vimentin in NHBE cells treated with CSE.

### Western blot analysis

Proteins were extracted from NHBE cells and mouse lung tissues. Western blot analyses were performed for the determination of protein expression levels. For densitometric analyses, protein bands on the blots were measured by the use of Eagle Eye II software.

### Quantitative real-time PCR

The levels of E-cadherin, ZO-1, Vimentin, N-cadherin and GAPDH mRNA in NHBE cells and mouse lung tissues were determined by qRT-PCR. The primers used were as follows: E-cadherin, forward 5′-TCGACACCCGATTCAAAGTGG-3′ and reverse 5′-TTCCAGAAACGGAGGCCTGAT-3′; ZO-1, forward 5′- GCAGCCACAACCAATTCATAG-3′ and reverse 5′-GCAGACGATGTTCATAGTTTC-3′; Vimentin, forward 5′-CCTTGACATTGAGATTGCCA-3′ and reverse 5′-GTATCAACCAGAGGGAGTGA-3′; N-cadherin, forward 5′-ATCAAGTGCCATTAGCCAAG-3′ and reverse 5′- CTGAGCAGTGAATGTTGTCA -3′; GAPDH, forward 5′-GCTGCCCAACGCACCGAATA-3′ and reverse 5′- GAGTCAACGGATTTGGTCGT-3′.

### Transfection of ERK5 lentiviral vectors

NHBE cells were stably transfected with lentiviral vector expressing ERK5 or its negative control vector according to manufacture's protocol. Following transfection, NHBE cells were exposed to CSE for 7 days and used for experiments.

### Mice and exposure to TS

Male BALB/c mice were purchased from the Animal Research Center of Nanjing Medical University. All mice were maintained and used in accordance with the guidelines of Animal Care and Welfare Committee of Nanjing Medical University. Ten mice were exposed to tobacco smoke in a smoking apparatus with target concentration of total particulate matter (TPM) of 85 mg/m^3^, or exposed to filtered air. Animals were exposed for 6 hours daily for 12 consecutive weeks. After the last exposure, mice were sacrificed and lung tissues were isolated for analysis.

### *In vivo* delivery of ERK 5 lentiviral vector

In a separate set of animal study, mice were intratracheally delivered with lentiviral vectors, as previously described [59–60]. Mice were randomly divided into four groups (*n* = 8 per group): filtered air group; TS-exposed group, mice were exposed to TS; TS+LV-control group, mice were delivered with lentiviral negative control vector and exposed to TS; TS+LV-ERK5 group, mice were delivered with lentiviral vector expressing ERK5 and exposed to TS. Following the complection of exposure, mice were sacrificed and lung tissues were collected for analysis.

### Statistical analysis

Statistical analyses were performed with SPSS 16.0. All data were expressed as mean ± standard deviation. One-way ANOVA was used for comparison of statistical differences among multiple groups, followed by the LSD significant difference test. For comparison between two groups, unpaired Student t test was used. A value of *p* < 0.05 was considered significantly different.

## SUPPLEMENTARY METHODS


