# Supplementary material for: ERK5 negatively regulates tobacco smoke-induced pulmonary epithelial–mesenchymal transition
Source: Oncotarget. 2015 Apr 17;6(23):19605–18. doi: 10.18632/oncotarget.3747 (PMC4637308; doi:10.18632/oncotarget.3747)
Supplement: Supplementary file 1 [file oncotarget-06-19605-s001.pdf]

SUPPLEMENTARY METHODS

Chemicals and reagents

Cell culture reagents were purchased from Gibco (Carlsbad, CA) unless otherwise stated. XMD8-92 was purchased from Tocris Bioscience (Bristol, UK). Lentiviral expression vector for ERK5 and negative control vector were purchased from GeneChem Co., Ltd (Shanghai, China). The primary antibodies for total ERK5, phosphorylated ERK5, phosphorylated c-Jun, phosphorylated c-Fos, FosB, JunD, E-cadherin, ß-catenin, N-cadherin, Vimentin, and Snail were obtained from Cell Signaling Technology (Beverly, MA). Antibodies for ZO-1 and GAPDH were from Santa Cruz Biotechnology (Santa Cruz, CA). Primers for E-cadherin, ZO-1, N- cadherin, Vimentin and GAPDH were synthesized by Invitrogen (Carlsbad, CA). Sources of other materials are noted accordingly in the text.

Cell culture

Normal human bronchial epithelial (NHBE) cells were purchased from XiangYa Central Experiment Laboratory (Changsha, China). Cells were cultured in RPMI 1640 Medium (Gibco, Carlsbad, CA) supplemented with 12% fetal bovine serum (Gibco, Carlsbad, CA), penicillin (100 U/ml) and streptomycin (100 µg/ml) (Gibco, Carlsbad, CA). The medium was changed every other day until cells reached 80% confluence. Experiments were performed with cells between passages 5-7. Cells cultures were maintained in a humidified incubator at 37°C with 5% CO_2.

Preparation of cigarette smoke extract

Cigarette smoke extract (CSE) was prepared daily immediately before use according to the reported method. Briefly, one filterless 3R4F Reference Cigarette (9 mg tar and 0.8 mg nicotine/cigarette) was combusted and the mainstream smoke was continuously drawn through a glass syringe containing 10 ml of fetal bovine serum-free RPMI 1640 that was pre-warmed to 37°C at a rate of 5min/cigarette. The resulting suspension was adjusted to pH 7.4 and then filtered through a 0.22-µm-pore size filter. The obtained solution was referred to as a 100% CSE solution and further diluted to the desired concentration with culture medium. Control solution was prepared with the same protocol, except that the cigarette was unlit. NHBE cells were exposed to various concentrations of CSE within 30 min of preparation.

Cytotoxicity assay

NHBE cells were seeded in 96-well plates at a density of 2×10^3 cells/well in 100 µl of medium. Twenty four hours later, the medium was replaced and cells were treated with 0%, 1%, 2%, 4%, 8% or 16% CSE for 7-14 days. Cell viability was then assessed using MTT assay. Ten microliter of methylthiazoletetrazolium (Promega Corporation, Madison, WI) assay solution (5 mg/mL) was added to each well and the plates were further incubated for 4 h at 37°C. Medium containing MTT was removed and precipitants were solubilized in DMSO. Absorbance was measured at 490 nm using a microplate reader (Titertek, Huntsville, AL). All measurements were performed in triplicate.

Transwell assays

Migration of CSE-treated NHBE cells was evaluated using Transwell chambers with 8-µm pore filters (Millipore, Billerica, MA). NHBE cells were trypsinized and seeded onto the upper chamber of the transwells (3×10^4 cells/well) in serum free medium. The lower chambers of the transwells were filled with medium containing 12% fetal bovine serum. The cells were then allowed to migrate for 24 h at 37°C, 5% CO_2. Nonmigrating cells on the upper surface of the filter were gently removed with a cotton swab. Cells migrating through the filter to the lower surface were fixed with 4% paraformaldehyde for 10 min and stained with 0.1% crystal violet for 5 min, and washed twice with PBS. Stained cells were visualized and photographed under a microscope, and the numbers of cells counted in three random fields were averaged.

For invasion assay, 3×10^4 NHBE cells were added to the upper chambers that had been coated with 50 µl of Matrigel (BD Biosciences, Franklin Lakes, NJ). Medium containing 12% fetal bovine serum was added to the lower chambers. Cells were incubated at 37°C for 24 h, and then non-invading cells were removed with cotton swabs. Cells invading through the filter were fixed, stained, viewed and counted in three randomly chosen fields.

Immunofluorescent staining

HBE cells were stained with rabbit E-cadherin and vimentin antibodies at 4°C overnight and then incubated with fluorescein isothiocyanate (FITC)-conjugated goat anti-rabbit secondary antibody (Leinco Technology, St Louis, MO) for 1 h. To stain the nuclei, 4′, 6-diamidino-2-phenylindole (DAPI, Sigma, St. Louis, MO) was added for 10 min. Images were captured using fluorescence microscopy (Zeiss, LSM700B, Germany).

Western blot analysis

HBE cells were washed twice with ice-cold PBS and scraped into 0.2 mL of lysis buffer [20 mM HEPES (pH 6.8), 5 mM EDTA, 10 mM EGTA, 5 mM NaF, 0.1 µg/mL okadaic acid, 1 mM dithiothreitol, 0.4 M KCl, 0.4% Triton

X-100, 10% glycerol, 5 μg/mL leupeptin, 50 μg/mL of phenylmethanesulphonylfluoride, 1 mM benzamidine, 5 mg/mL aprotinin and 1 mM sodium orthovanadate]. In animal study, lung tissues were homogenized in a lysate buffer (5 mmol/L EDTA, 50 mmol/L Tris, 1% SDS, pH 7.5, 10 μg/mL aprotinin, 1% sodium deoxycholate, 1% NP-40, 1 mM PMSF, 1% Triton-X 100, and 10 μg/mL leupeptin) and then centrifuged at 4°C for 20 min. Protein concentrations were measured with the BCA Protein Assay (Pierce, Rockford, IL). Fifty micrograms of proteins were fractionated by electrophoresis through 7.5–10% SDS-PAGE and were transferred to PVDF membrane (Millipore, Billerica, MA). The membranes were blocked with 5% defatted milk and subsequently probed with primary antibody overnight at 4°C, and then incubated with horseradish peroxidase-conjugated secondary antibody. GAPDH served as the loading control. For densitometric analyses, protein bands on the blots were measured by the use of Eagle Eye II software.

Quantitative reverse transcriptase-polymerase chain reaction

Total RNA was isolated by RNAiso Plus according to the manufacturer the manufacturer's instructions (TaKaRa, Japan) . Two micrograms of total RNA was reverse transcribed into cDNA using AMV Reverse Transcriptase (Promega, Madison, WI). qRT-PCR was performed using the Power SYBR Green Master Mix (TaKaRa, Japan) and an ABI 7300 real-time PCR detection system (Applied Biosystems, CA). The primers used were as follows: E-cadherin, forward 5′-TCGACACCCGATTCAAAGTGG-3′ and reverse 5′-TTCCAGAAACGGAGGCCTGAT-3′; ZO-1, forward 5′-GCAGCCACAACCAATTCATAG-3′ and reverse 5′-GCAGACGATGTTCATAGTTTC-3′; Vimentin, forward 5′-CCTTGACATTGAGATTGCCA-3′ and reverse 5′-GTATCAACCAGAGGGAGTGA-3′; N-cadherin forward 5′-ATCAAGTGCCATTAGCCAAG-3′ and reverse 5′-CTGAGCAGTGAATGTTGTCA-3′; GAPDH, forward 5′-GCTGCCCAACGCACCGAATA-3′ and reverse 5′-GAGTCAACGGATTTGGTCGT-3′. All of the primers were synthesized by Invitrogen (Carlsbad, CA). The levels of mRNA expression for each gene were normalized by its respective GAPDH. Fold changes in gene expression were calculated by a comparative threshold cycle (Ct) method using the formula $2^{-(\Delta\Delta Ct)}$.

Transfection of ERK5 lentiviral vectors

NHBE cells were stably transfected with lentiviral expression vector for ERK5 or its negative control vector according to manufacture's protocol. Briefly, cells were cultured in RPMI Medium 1640 with 12% fetal bovine serum, and infected with lentiviral vector at multiplicity of infection (MOI) of 2.5, 5, 10, 25 and 50. The infection medium was removed and replaced by fresh medium after overnight incubation. The transfection efficacy was determined three days later by Green fluorescent protein (GFP) fluorescence imaging using fluorescent microscopy. Transfection assays were accomplished at a MOI of 25, the optimal infection efficiency. Seventy two hours after transfection, NHBE cells were exposed to CSE for 7 days and used for experiments.

Mice and exposure to TS

Eight-week-old male BALB/c mice weighing 18-22 g were purchased from the Animal Research Center of Nanjing Medical University. All mice were allowed to acclimate for 1 week prior to the onset of experimental exposure. The mice were housed in polypropylene cages, maintained on a 12-hour light/dark cycle, 22 ± 0.5°C room temperature, 40–60% relative humidity, and free access to water and food. Animals were handled in accordance with the recommendations in the guidelines of the Animal Care and Welfare Committee of Nanjing Medical University. The study protocol was approved by the Committee on the Ethics of Animal Experiments of Nanjing Medical University.

Mice were exposed to tobacco smoke in a smoking apparatus. TS was generated by a smoke machine that smoked the cigarettes and pumped the mainstream cigarette smoke from burning cigarettes at a constant rate (each cigarette took 5 min to burn out). The smoke was delivered to whole-body exposure chambers with target concentration of total particulate matter (TPM) of 85 mg/m^3. Animals were exposed for 6 hours daily for 12 consecutive weeks. Animals in the control group were exposed to filtered, conditioned air. Ten mice were randomly assigned into each group. The exposures were monitored and characterized as the followings: carbon monoxide (16.31 ± 2.16 ppm), TPM (0 mg/m^3) for the control group; carbon monoxide (172.07 ± 19.38 ppm), TPM (85.28 ± 4.81 mg/m^3) for TS exposure group. After the last TS exposure, mice were sacrificed and lung tissues were isolated, frozen and stored at –80°C until analysis.

In vivo delivery of ERK 5 lentiviral vector

In a separate set of animal study, mice were intratracheally delivered with lentiviral vectors, as previously described. In brief, the neck was extended and cleaned with a chlorhexidine solution. A small midline incision was made with a sterile scalpel to expose the trachea. 4 × 10^7 vector particles in a final volume of 50 μL of phosphate-buffered saline were instilled into the trachea with a 27-gauge needle. Upon completion of the instillation, the skin and fascia were closed in one layer with interrupted sutures (4-0 Vicryl). Mice were randomly divided into four groups (n = 8 per group): filtered air group; TS-exposed group, mice were exposed to TS;

TS+LV-control group, mice were delivered with lentiviral negative control vector and exposed to TS; TS + LV-ERK5 group, mice were delivered with lentiviral vector expressing ERK5 and exposed to TS. The intratracheal delivery of lentiviral vectors was performed every four weeks and mice were exposed to filtered air or TS with a target concentration of 85 mg/m^3 TPM for 12 weeks. Following the complection of exposure, mice were sacrificed and lung tissues were collected for analysis.

Statistical analysis

Statistical analyses were performed with SPSS 16.0. All data were expressed as mean ± standard deviation. One-way ANOVA was used for comparison of statistical differences among multiple groups, followed by the LSD significant difference test. In case of comparison between two groups, unpaired Student *t* test was used. A value of $p < 0.05$ was considered significantly different.
